# Effectiveness of a Flossing Protocol and Manual Therapy in Improving the Clinical and Functional Status of Subjects with Recurrent Ankle Sprains; A Double-Blind Randomized Clinical Trial

**DOI:** 10.3390/medsci13030149

**Published:** 2025-08-20

**Authors:** Mario Bermúdez-Egidos, Raúl Pérez-Llanes, Rubén Cuesta-Barriuso

**Affiliations:** 1 Department of Physiotherapy, University of Murcia, 30120 Murcia, Spain; rperez@um.es; 2 InHeFis Research Group, Instituto Asturiano de Investigación Sanitaria (ISPA), 33011 Oviedo, Spain; cuestaruben@uniovi.es; 3 Department of Surgery and Medical-Surgical Specialties, University of Oviedo, 33006 Oviedo, Spain

**Keywords:** sprain, flossing, manual therapy, range of motion, pressure pain threshold, stability

## Abstract

**Introduction**: Recurrent ankle sprains can lead to chronic ankle instability. The flossing technique aims to modify the function and characteristics of fascial tissue. The objective was to evaluate the effectiveness of flossing and sliding techniques in improving subjects with previous ankle sprains. **Methods**: Randomized, double-blind clinical study with a follow-up period. Twenty-six subjects were assigned to two study groups: experimental (flossing technique and passive manual therapy techniques) and placebo control group (flossing technique without compression and manual therapy techniques without sliding). The intervention lasted three weeks, with two sessions per week. The study variables were dorsiflexion under load (Leg Motion^®^), ankle mobility under unloaded conditions (goniometer), pressure pain threshold (algometer), and stability (Rs Scan^®^ pressure platform). Three measurements were taken: pre-treatment (T0), post-treatment (T1), and after 3 weeks of follow-up (T2). **Results**: There were significant intergroup differences in dorsiflexion under load (F = 4.90; *p* = 0.02). Range of motion in plantar flexion without load (F = 3.78; *p* = 0.04), in the ellipse area (F = 4.72; *p* = 0.01), left stability (F = 3.74; *p* = 0.03), and right stability (F = 3.73; *p* = 0.03) without visual support. **Conclusions**: A physiotherapy protocol using flossing and manual sliding therapy can increase loaded dorsal flexion in young adults with previous ankle sprains. This intervention can also improve ankle plantar flexion under unloaded conditions. The area of the ellipse without visual support can improve in young adults with a history of ankle sprains following a program of flossing and manual therapy.

## 1. Introduction

Ankle injuries account for 15–30% of sports injuries [1]. Ankle sprains are ligament injuries caused by stretching or tearing of the ankle joint. Ankle sprains manifest as chronic pain, decreased range of motion, inflammation, and instability [2]. As a result, in sports, there is a decrease in athletic performance [3].

The most common cause of these injuries is high- or low-impact trauma. Depending on the positional mechanism, we find lateral sprains (inversion and dorsal flexion), medial sprains (eversion), and syndesmotic sprains (external rotation or dorsal flexion) [4]. These sprains account for 16–40% of ankle injuries [5].

The most common sprains occur with lateral mechanisms and are more frequent in sports such as basketball, American football, and soccer [6]. Eighty-five percent of ankle ligament injuries are usually caused by an inversion movement of the ankle. This injury to the lateral collateral ligaments of the ankle manifests itself with pain, swelling, and decreased dorsiflexion of the ankle [7].

The risk factors for lateral ankle sprains can be intrinsic and extrinsic. The main intrinsic risk factors for sprains are limited range of motion in dorsiflexion [8], proprioceptive alterations [9], and poor postural control [10]. Another factor, which cannot be modified, is gender, with a higher predisposition reported in women [11]. The main extrinsic risk factor is the type of sport practiced, with a high prevalence of injury in activities such as basketball, volleyball, field sports, and climbing [12]. There are three degrees of sprain: grade I (mild), grade II (moderate), and grade III (total injury, most severe) [13].

Dorsiflexion is important during walking and shock absorption [14]. Limitations of this movement can alter the biomechanics of the lower limb [9], reducing the degree of knee flexion under load and favoring valgus knee translation during landing and squatting. This increases the risk of injury. Chronicity of this ligament injury can lead to chronic ankle instability [15].

The flossing technique involves firmly and evenly wrapping an elastic band around a joint with the aim of modifying the function and characteristics of the fascial tissue [16]. These changes can be caused by increased perfusion and reduced myofascial stiffness resulting from the compression exerted by the band. This technique has been shown to be effective in improving ankle range of motion in professional basketball players [17]. Similarly, in young athletes, its application has shown changes in jump performance, muscle strength, and balance [18].

Our study hypothesis was that combining flossing with manual therapy and active exercises could cause changes in ankle joint range of motion, loading and unloading, pressure pain threshold, and stability in subjects with a history of ankle sprains. The main objective of this study was to evaluate the efficacy of flossing and passive manual therapy techniques in the clinical improvement of subjects with previous ankle sprains.

## 2. Materials and Methods

### 2.1. Study Design

Randomized, double-blind clinical study with a follow-up period.

### 2.2. Ethical Considerations

The study was designed and conducted in accordance with the criteria defined in the Declaration of Helsinki. The individuals included in the study were informed about the objectives, possible risks, and benefits of the study. All participants signed the informed consent document.

The Ethics Committee of the Catholic University of San Antonio in Murcia approved the research project (ID: CE-022306; date: 24 February 2023). The research project was registered in an international clinical trial registry (www.clinicaltrials.gov; ID: NCT05859256; date: 5 May 2023).

### 2.3. Participants

The inclusion criteria for participating in the study were: subjects over 18 years of age; of both sexes; having suffered at least two ankle sprains prior to the study; lacking chronic or degenerative musculoskeletal pathologies in the ankle or knee; and engaging in regular physical activity. Exclusion criteria were having suffered an ankle sprain in the 4 months prior to the study or receiving other physiotherapy treatment on the lower limbs during the study period.

Participants were recruited at the Giro Deporte y Salud Centre in Murcia and the Catholic University of Murcia. The study was conducted between May and August 2023.

### 2.4. Intervention

The interventions in both groups lasted 3 weeks, with 2 sessions per week, each lasting 10 min [19].

**Figure 1:**
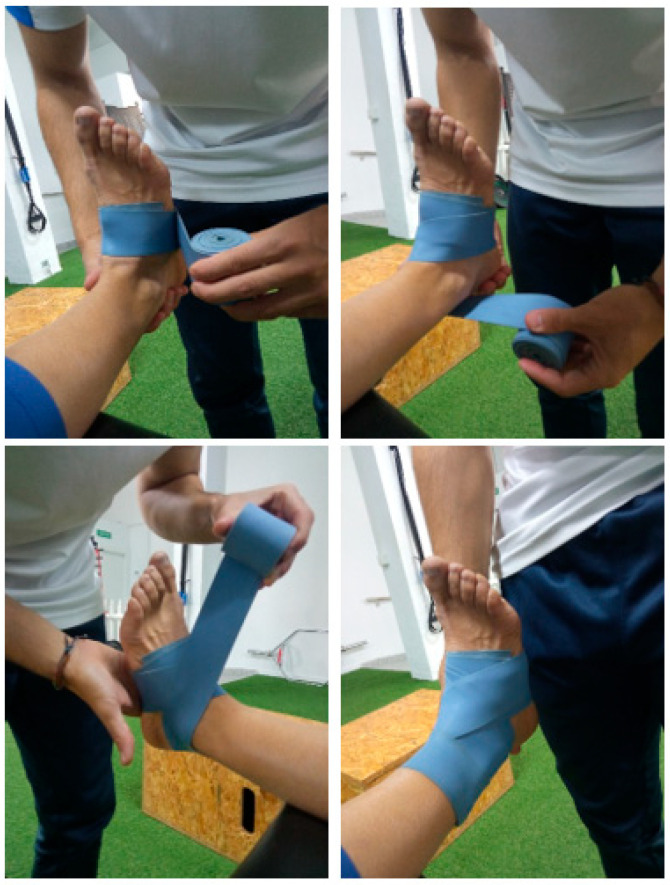
Protocol for placing floss during the intervention carried out in the study.

In the experimental group, with the patient barefoot, the session began with a warm-up without the elastic band, consisting of three active exercises: 1 min of walking in a straight line, 15 heel raises with weight bearing, and 15 dorsiflexion of the ankle of the lower limb to be treated in a standing position. The elastic band or flossing tape was then applied, starting transversely at the metatarsals with two passes in a caudocranial direction, from the dorsum of the foot to the first toe and passing through the sole of the foot to the fifth toe, returning to the dorsum. Once the two turns were completed, it was placed in a figure-8 shape, passing through both malleolar regions and wrapping around the Achilles tendon, descending caudally in an oblique direction to the medial part of the foot. The elastic band was passed under the sole of the foot again to start the same route, making a total of three figure-of-eight turns. The excess band was placed on the subject’s leg to secure it [20]. The elastic band used was blue and measured 5 cm wide, 200 cm long, and 1.3 mm thick. Figure 1 shows the placement of the flossing.

Once the flossing was in place, the patient repeated the active warm-up. Upon completion, manual techniques were performed according to the Kaltenborn method. With the patient in the supine position, leaving the foot and ankle off the table and the distal tibiofibular area of the leg fixed against it, the physiotherapist grasped the calcaneus by its lateral edge, generating slight traction. With the contralateral hand, on the medial side of the ankle, the physiotherapist placed their thumb on the talus. The direction of mobilization was dorsal to improve dorsal flexion of the ankle [21].

In the second technique, with the subject in the prone position and the foot outside the table, the physiotherapist slid the cranial hand in a ventral direction while the other hand held the talus and tarsus, pulling slightly distally. The technique consists of applying a forward pushing force, generating joint sliding to improve the range of plantar ankle movement [21]. At the end of the manual techniques, the flossing was removed in the opposite direction to its placement, with the leg raised and quickly, repeating the warm-up exercises.

The subjects included in the placebo control group received the same intervention, with the same frequency and exercises as those assigned to the experimental group. However, the elastic band was placed without any tension to avoid compression. On the other hand, when performing the passive sliding techniques, the physiotherapist placed the subjects in the same position, performing the same movements, but without causing any joint sliding.

### 2.5. Outcomes Measurements

The primary dependent variable was dorsiflexion under load. Secondary variables were ankle range of motion in plantar and dorsal flexion, pressure pain threshold, and foot and ankle stability.

- The range of motion of the ankle in dorsiflexion under load was measured using the Leg Motion^®^ system (CheckyourMOtion, Albacete, Spain) [22]. The patient stood on the platform with their hands on their hips. The second toe and the center of the heel were aligned with the longitudinal line, without stepping on the transverse line (0 cm). With the other foot behind the platform and without losing contact with the floor, the subject shifted their weight to the leg being evaluated, flexing the knee, and without lifting the heel off the contact surface. This instrument has shown high reliability (ICC = 0.96–0.98) [23].
- The range of motion of the ankle in unweighted bearing, dorsiflexion, and plantar flexion was assessed with a universal goniometer. The patient was placed in the supine position with the knees extended and relaxed. The fixed arm was aligned with the diaphysis of the fibula, the mobile arm was aligned with the head of the fifth metatarsal, and the axis of the goniometer was adjusted below the lateral malleolus [24]. Two measurements were taken for each movement, and the mean value was used as the result [25]. This instrument has shown high reliability (ICC = 0.93 and ICC = 0.91 for plantar and dorsal flexion, respectively) [26].
- The pressure pain threshold was measured using a pressure algometer (model Wagner FDIX. Wagner Instruments, Riverside, CT, USA) [27]. The threshold was measured in both lower limbs by placing the algometer below the medial and lateral malleoli [28]. The evaluator applied constant pressure until the subject perceived the pressure as uncomfortable. This instrument has shown high reliability (ICC = 0.98–0.99) [29].
- Standing and ankle stability were measured using a biomechanical analysis of balance and gait employing an Rs Scan^®^ pressure platform and FootScan^®^ pressure measurement system. This scientific version of the biomechanical examination device measures plantar pressure using an X-Y matrix of sensitive resistive pressure sensors that are scanned sequentially. The system records pressure data when the subject is standing or walking on the platform. The measurements were taken using the basic 0.5 m platform with 4096 resistive sensors and a data acquisition frequency of 300 Hz. This instrument measures the subject’s plantar contact when standing on or walking over the platform. Gait was assessed by walking on the platform, and static balance was assessed for 30 s with eyes open and closed [30].

Three measurements were taken during the study: baseline (T0), post-treatment (T1), and after a 3-week follow-up period (T2).

Before recruiting the subjects, a pilot study was conducted to measure intra-observer reliability in the dependent variables. High reliability was obtained in plantar flexion (ICC = 0.98), pain threshold in the medial malleolus (ICC = 0.83), and stability (ICC = 0.83). In dorsal flexion (ICC = 0.60) and pain threshold in the lateral malleolus (ICC = 0.74), intra-observer reliability was moderate.

### 2.6. Sample Size

Prior to recruiting the subjects, the sample size was calculated using the statistical package G*Power (version 3.1.9.2; HeinrichHeine-Universität, Düsseldorf, Germany). The study by Stevenson et al. [31] was used as a reference, which identified a moderate effect size (d = 0.68) for the use of flossing on ankle range of motion. With an alpha level (type I error) of 0.05 and a statistical power of 95% (1-β = 0.95), a sample size of 22 patients was obtained. Considering the probability of dropout during the study, 15% more subjects were recruited.

### 2.7. Randomization and Blinding

The randomization of subjects who met the selection criteria to the study groups was carried out using a permuted block system. Participants were randomly allocated to the experimental or control group using block randomisation with a fixed block size of four, to ensure balanced allocation throughout recruitment. No stratification based on baseline characteristics (e.g., age, BMI, number of previous sprains) was applied; blocks served purely to regulate group sizes during assignment. The final group sizes (n = 12 and n = 14) reflect a slight imbalance due to participant dropouts and the final block not being fully completed at the end of recruitment.

The person responsible for assigning subjects to one study group or the other was blinded to the study objectives and the characteristics of the study groups.

The physiotherapist responsible for conducting the three assessments in this study was blinded to the assignment of subjects to each of the study groups. Similarly, the participants included in the study were blinded to their assignment to each of the study groups and did not know whether they were receiving the experimental intervention or the placebo control.

### 2.8. Statistical Analysis

Data analysis was performed using SPSS 19.0 software (IBM SPSS Statistics for Windows; IBM Corp., New York, NY, USA).

Intraobserver reliability was analyzed using the two-factor random intraclass correlation coefficient. For comparisons between groups at each assessment point, the Mann–Whitney U test was applied to ordinal and non-normally distributed data, as determined by the Shapiro–Wilk test. Intergroup differences in qualitative variables were calculated using Fisher’s exact test. The sample was described using the median and interquartile range.

Although some data were not normally distributed, F tests are robust in terms of type I error and, regardless of the conditions manipulated, are considered a valid option for non-parametric distributions [32].

The intergroup effect was calculated using the repeated measures ANOVA test. A two-way repeated measures ANOVA (group × time) was used to examine the effects of the intervention on the dependent variables. Although some variables did not follow a perfectly normal distribution, this analysis was considered appropriate given the method’s robustness to moderate violations of normality, particularly when group sizes are similar, and no extreme outliers are present. Greenhouse–Geisser corrections were applied where required [32,33]. These statistical procedures have been reported in accordance with the robustness of the repeated measures ANOVA and non-parametric testing guidelines. The Greenhouse-Geisser correction was used when Mauchly’s test rejected (*p* < 0.05) sphericity. Post hoc comparisons and pairwise analysis were performed using the Bonferroni correction to control for Type I error in multiple comparisons. The effect size of the F tests was calculated using the eta-squared coefficients (η^2^_p_) [34]. A post hoc power analysis was performed to assess the sensitivity of the design to detect significant effects using G*Power 3.1 software (Heinrich-Heine-Universität, Düsseldorf, Germany). A repeated measures model with two groups (intervention and placebo control) and three assessment points (baseline, post-treatment, and follow-up) was specified. The analysis was performed based on the effect size (η^2^_p_) observed in the Time*Group interaction for the dependent variables, the sample size, and a significance level of α = 0.05.

To complement the statistical analysis with clinical interpretation, we also calculated the minimum detectable change at the 95% confidence level (MDC_95_) for each continuous variable. These values were derived from inter-rater reliability (ICC) and the standard deviation of the baseline scores. In the absence of established anchor-based thresholds, MDC_95_ was adopted as a conservative estimate of the minimal clinically important difference (MCID). This approach is consistent with published recommendations in musculoskeletal outcomes research and allows identification of changes that exceed measurement error and may reflect meaningful clinical improvement [35].

In this study, an intention-to-treat analysis was performed. According to the a priori sample size calculation parameters, statistical significance was set at α < 0.05 for a 95% confidence interval (CI).

## 3. Results

### 3.1. Participants Data

**Figure 2:**
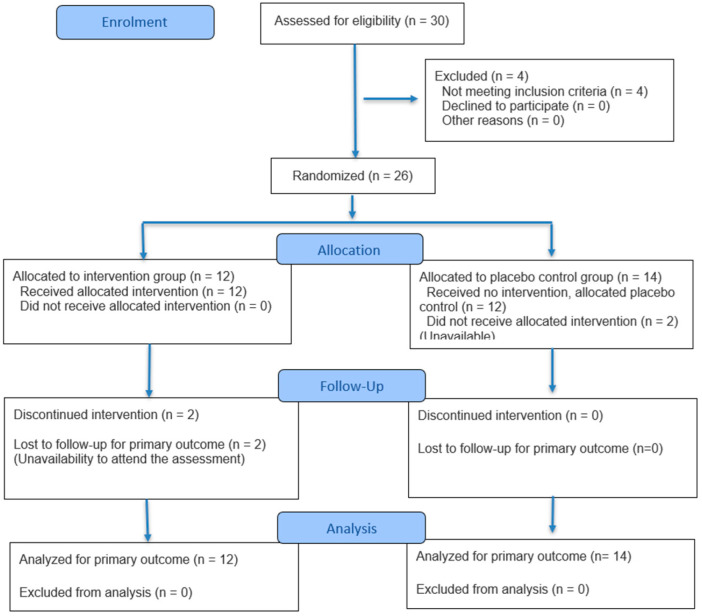
Study flow chart.

Of the 30 subjects invited to participate in the study, 26 met the selection criteria and agreed to take part. During the experimental phase, two participants withdrew from the study. In the follow-up assessment, two subjects were unable to participate for various reasons. Finally, 22 people completed the study. Figure 2 shows the flow chart of the study.

### 3.2. Safety of the Intervention

None of the subjects included in the study, in any of the groups, presented clinical complications or adverse effects as a direct or indirect consequence of the administration of the interventions carried out. The withdrawal of subjects who did not complete the study was due to personal and time constraints, and the intervention or its effect was not a direct cause of this.

### 3.3. Descriptive Analysis

The median age of participants was 27.5 (interquartile range: 7) years, with a body mass index of 24.35 (RI: 2.9) kg/m^2^. Most subjects were male (65.4%), with a median of 1 (IQR: 2) previous ankle sprains. Regarding regular sports practice, the subjects included in the study performed a median of 6 (IQR: 3) hours of exercise per week. Regarding postural stability, assessed via the principal axis of the ellipse at baseline, no statistically significant differences (*p* = 0.66) were observed between the experimental and placebo control groups. The experimental group demonstrated a median value of 4.0 mm (IQR: 2.0), compared to 3.5 mm (IQR: 4.25) in the control group. These findings indicate comparable baseline levels of postural control across groups. Table 1 shows the descriptive analysis of the participants according to the study group. Table 2 shows the central tendency and dispersion statistics of the study variables throughout the different evaluations carried out during the study.

### 3.4. Analysis of Repeated Measures

In the analysis of the intragroup effect, there were statistically significant differences (*p* < 0.001) in the variables dorsal flexion under load (F_[1.26; 63.16]_ = 43.73; η^2^_p_ = 0.46), dorsal (F_[2; 100]_ = 17.12; η^2^_p_ = 0.25) and plantar flexion in unloaded conditions (F_[1.37; 68.55]_ = 18.46; η^2^_p_ = 0.27), and pain threshold to pressure on the medial (F_[1.54; 77.34]_ = 20.62; η^2^_p_ = 0.29) and external malleolus (F_[1.67; 83.59]_ = 10.41; η^2^_p_ = 0.17). Regarding the assessment of stability. There were significant changes in the minimum (F_[2; 48]_ = 3.87; *p* = 0.02; η^2^_p_ = 0.13) and maximum (F_[1.59; 38.21]_ = 3.80; *p* = 0.04; η^2^_p_ = 0.13) anterior movement (y-axis) with eyes open, and the area of the ellipse without visual support (F_[2; 48]_ = 4.72; *p* = 0.01; η^2^_p_ = 0.16).

**Figure 3:**
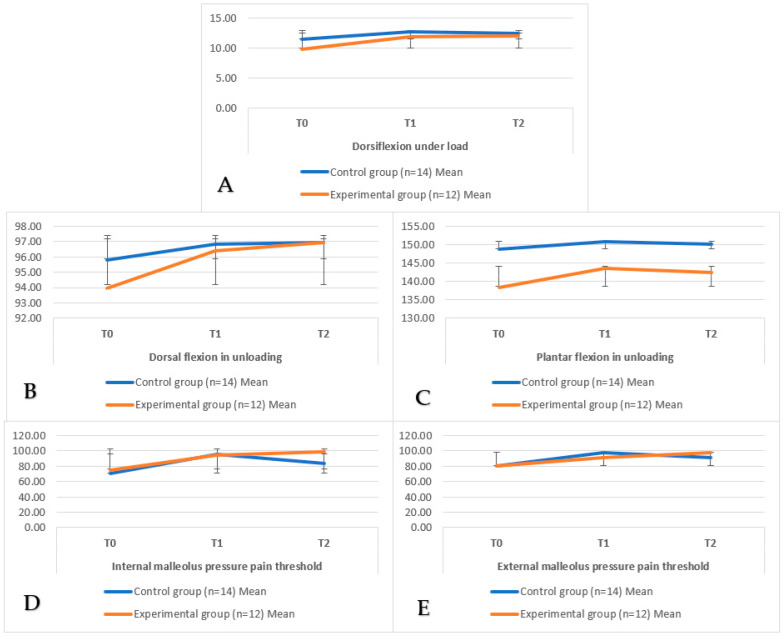
(**A**) Dorsiflexion under load; (**B**) Dorsal flexion in unloading; (**C**) Plantar flexion in unloading; (**D**) Internal malleolus pressure pain threshold; (**E**) External malleolus pressure pain threshold.

In the analysis of repeated measures, we observed statistically significant differences in the time*group interaction in the dorsal flexion under load (F = 4.90; *p* = 0.02; η^2^_p_ = 0.08), and plantar flexion under no load (F = 3.78; *p* = 0.04; η^2^_p_ = 0.07). In the stability assessment, there were statistically significant changes in the ellipse area (F_[2;48]_ = 4.72; *p* = 0.01; η^2^_p_ = 0.16). There were no statistically significant differences in the other variables. Table 3 and Table 4 show the results of the repeated measures analysis and the pairwise comparison analysis, respectively. To illustrate the evolution of the outcome measures over time between groups, interaction plots for the variables with significant group × time effects are shown in Figure 3.

### 3.5. Analysis Minimum Detectable Change

The standard error of measurement (SEM) and the minimum detectable change at the 95% confidence level (MDC_95_) varied across the measured variables. The lowest MDC_95_ was observed for postural stability with eyes open (0.07 mm), while the highest corresponded to the pressure pain threshold at the internal malleolus (28.7 N). Regarding the percentage of participants who exceeded the MDC_95_ threshold—indicating a clinically relevant improvement—a greater proportion was found in the experimental group (EG) across most variables. A total of 83.33% of the EG showed improvement in dorsiflexion under load, compared to 35.7% in the control group (CG). Similar trends were observed in dorsal flexion in unloading (50% in EG vs. 28.6% in CG) and plantar flexion in unloading (41.7% in EG vs. 28.6% in CG). In contrast, postural stability variables showed lower rates of improvement overall, particularly in the eyes-closed condition. Table 5 summarizes the inter-rater reliability results, as well as the estimated standard error of measurement (SEM) and minimum detectable change at the 95% confidence level (MDC_95_).

## 4. Discussion

This study aimed to evaluate the effectiveness of an active exercise treatment protocol with the application of flossing and manual therapy techniques on the ankle in improving range of motion, pressure pain threshold, and static and dynamic stability in subjects with a history of ankle sprain. In the intergroup analysis, there were statistically significant differences in dorsiflexion under load, plantar flexion under unload, and stability without visual support.

The flossing technique, based on the application of an elastic band to musculoskeletal regions, can generate physiological reactions that promote recovery and functionality. These improvements may be due, among other factors, to changes in fascial flexibility, which is necessary for proper tissue fluidity during joint movement. In addition, the partial restriction of blood flow caused by the elastic band promotes the elimination of waste products and the delivery of nutrients to the tissue [36].

Driller et al. [20] observed an improvement in ankle dorsiflexion under load after applying a flossing protocol. Flossing causes a shearing effect on the fascia [37]. Similarly, it increases joint lubrication after blood perfusion following ischemia produced by the application of the band [20]. These results coincide with the changes observed in our study, where there was a statistically significant improvement in dorsiflexion under load.

Regarding the improvement in range of motion in plantar flexion observed in our study, the facilitation of anterior-posterior talar sliding should be considered [38]. The flossing technique causes myofascial compression that promotes joint decoupling, facilitating the application of mobilization techniques with less pain and restriction [36]. On the other hand, the mechanical muscle effect that can facilitate an increased range of motion should be considered. The fascial shearing effect and blood flow restriction caused by the flossing technique may facilitate an increase in the concentration of growth hormones and catecholamines. In this way, it can induce an improvement in strength and the quality and effectiveness of muscle contraction [37]. An increase in spinal excitation can improve performance, as afferent signals from the neuromuscular spindles participate in muscle contraction [38].

No intergroup differences were observed in the pressure pain threshold. The active warm-up exercises performed in both protocols involve active joint mobilization, facilitating stretching of the gastrocnemius and soleus muscles [39]. In line with our results, it has been observed [40] that in subjects with recurrent ankle sprains and functional instability, the application of two active mobilization interventions, combined or not with manual techniques, leads to improvements in pain intensity and pressure threshold. Contrary to our findings, Shin et al. [38] observed how an intervention based on passive manipulation techniques, together with warm-up and active exercises, improved the pressure pain threshold, compared to the absence of manual techniques. The compression force applied by the band stimulates mechanoreceptors and free nerve endings capable of modulating pain and benefiting proprioceptive inputs [41].

In our study, we observed changes throughout the interventions in both groups (intra-group effect) in anterior movement, in the axis, and with visual support. This decrease in static sway in both groups may be due to the contribution of synergistic stimuli on the ligamentous and capsular proprioceptors of the ankle. This stimulation may be complemented by the relaxation of shortened muscles to promote greater proprioceptive input and increased muscle activity [38]. However, in the stability variables measured with and without visual support, no intergroup differences were observed in posterior movement (x-axis), anterior movement (y-axis), or ellipse area.

The design of the placebo intervention aimed to replicate non-specific treatment components—such as therapist contact, tactile input, and passive joint handling—while removing the biomechanical action of the experimental techniques. In the placebo group, the flossing band was applied without tension to eliminate compression, and the manual techniques were executed without producing effective joint sliding. Although these procedures may not have been entirely inert, prior evidence suggests that touch and passive movement alone can evoke physiological and analgesic responses. These effects, although non-specific, may influence participant perception and therapeutic outcomes [42]. Placebo-controlled trials in manual therapy have highlighted the importance of matching sensory input and therapist interaction in order to isolate specific treatment effects [43]. In our study, this design was essential to ensure participant blinding and internal validity while acknowledging the possibility of a minimal placebo response induced by somatosensory stimulation.

### Limitations of the Study

This study has several limitations that should be considered before generalizing the results. Although some studies [20] detail the pressure applied to determine the optimal pressure of the elastic band, this calculation was not performed in our study. The implementation of this measure would have provided more information on the effects of the vascular occlusion caused, since pressures ≥200 mmHg can cause neuromuscular inhibition, and pressures above ≥230 mmHg can reduce the soleus H reflex [44]. In addition, no objective method (e.g., manometer or pressure sensors) was used to quantify the compression applied during the intervention, nor was participants’ tolerance recorded. This limits the reproducibility of the protocol and complicates comparisons with other studies. Future research should incorporate objective pressure measurement tools and report both the applied values and the participants’ perceived tolerance.

Similarly, the available scientific evidence on the physiological mechanisms induced by the flossing technique should be considered. The changes mentioned in some studies are blood revascularization after removal of the elastic band, hormonal and catecholamine responses, and changes in muscle activation. Therefore, the scientific evidence on these types of changes and the influence of these techniques on injury rehabilitation should continue to be expanded [11].

Although the sample size required for this study was calculated a priori using G*Power software and based on previously reported effect sizes, the final number of analysed participants (n = 22) may have reduced the statistical power and limited the generalisability of the results. Moreover, the relatively high dropout rate observed during the intervention and follow-up phases suggests that future studies should account for a greater attrition margin and implement procedures to systematically document and analyse the reasons for participant withdrawal. While an intention-to-treat analysis was performed, caution should be exercised when extrapolating the findings to broader populations.

Regarding the magnitude of the intervention, this study only explored the results after a 3-week treatment period, and follow-up assessments were limited to three weeks after the end of the intervention. Although the findings revealed statistically significant improvements in some variables, the short duration of both the treatment and follow-up periods prevents conclusions about the persistence or durability of the effects over time. In contrast, other studies have applied interventions over longer timeframes (e.g., 6–8 weeks), and follow-up periods of several months (e.g., 3–6 months) are often recommended to assess long-term clinical relevance. Future research should therefore consider extending both the intervention duration and the follow-up period to evaluate the sustained effects of these therapies more comprehensively.

Additionally, although inclusion criteria ensured that participants engaged in regular physical activity and had not received physiotherapy on the lower limbs within the past four months, we did not collect detailed data on the specific type of sport practised, the exact volume and intensity of physical activity during the study period, or previous therapeutic exposures beyond the exclusion window. These uncontrolled variables may have acted as potential confounders and influenced individual responsiveness to the intervention. Future studies should systematically record and control for these factors to improve the internal validity and interpretability of results.

## 5. Conclusions

A physiotherapy protocol involving flossing and manual therapy with sliding can improve dorsiflexion under load in subjects with previous ankle sprains. The combination of flossing and joint sliding can improve ankle plantar flexion at rest in people with previous ankle sprains. Left and right stability variables and the area of the ellipse without visual support can improve in young adults with previous ankle sprains.

## Figures and Tables

**Table 1:** Descriptive analysis, median (and interquartile range), of the subjects included in the study.

Variables	Experimental Group (n = 12)	Placebo Control Group (n = 14)	Sig.
Age (years)	28.0 (6.50)	25.50 (9.75)	0.41 ^†^
Weight (kg)	75.5 (24.50)	73. (23.00)	0.48 ^†^
Height (m)	1.75 (0.14)	1.74 (0.13)	0.17 ^†^
Body mass index (kg/m^2^)	24.1 (5.70)	24.35 (2.90)	0.88 ^†^
Previous sprains (number)	1.50 (1.75)	1.0 (2.0)	0.30 ^†^
Time elapsed since last sprain (months)	9.0 (7.75)	11.50 (6.0)	0.42 ^†^
Regular exercise (h/week)	5.0 (4.25)	6.50 (3.50)	0.39 ^†^
Postural stability (mm)	4.0 (2.0)	3.50 (4.25)	0.66 ^†^
	n (%)		
Gender (Male/Female)	8/4 (66.7/33.3)	9/5 (64.3/35.7)	1.00 ^‡^

Sig.: signification; n: number of subjects; %: percentage. ^†^ Mann-Whitney U test; ^‡^ Fisher’s exact test.

**Table 2:** Statistics of central tendency, median, and dispersion (interquartile range) of the study variables, in both groups.

Variables	Experimental Group			Placebo Control Group		
	T0	T1	T2	T0	T1	T2
Dorsiflexion under load (cm)	9.75 (4.88)	12 (4.75)	12 (5)	10.75 (5.25)	12.75 (5.87)	12.5 (5.87)
Dorsal flexion in unloading (degrees)	93 (8.5)	96 (7.5)	96.5 (9.5)	96 (5.75)	98 (5.75)	97.5 (6)
Plantar flexion in unloading (degrees)	140 (19)	143 (16)	141 (12.75)	146.5 (13)	149.5 (15)	148 (12.75)
Internal malleolus pressure pain threshold (Newton)	71.52 (26.75)	94.12 (59.67)	99.85 (64.2)	66.87 (38.17)	93.25 (58.89)	81.35 (47.02)
External malleolus pressure pain threshold (Newton)	75.5 (30.21)	96.1 (48.13)	100.47 (37.94)	78.07 (51.72)	97.52 (77.81)	84.62 (43.06)
Min-X with open eyes (mm)	184 (21.75)	173.5 (29.75)	184.5 (22)	180.5 (21.25)	182.5 (20)	174.5 (26.75)
Min-Y with open eyes (mm)	248.5 (14)	239.5 (13.75)	241.5 (14)	249 (16.5)	241.5 (14.25)	247 (21.5)
Max-X with open eyes (mm)	195 (19.75)	184 (31.25)	193 (30)	191 (18.75)	193 (24.25)	183.5 (23.75)
Max-Y with open eyes (mm)	254 (15.25)	242.5 (19)	244.5 (14.5)	252 (16)	245.5 (15.5)	251.5 (21.5)
Distance covered with open eyes (mm)	45.0 (28.75)	48.5 (56.25)	46.0 (21.5)	51.5 (38.75)	48.5 (31.5)	50.50 (30.5)
Area with open eyes (mm^2^)	3.0 (9.5)	2.50 (7.75)	3.0 (2.0)	4.0 (4.0)	4.0 (3.5)	3.00 (3.75)
Min-X with closed eyes (mm)	170 (22.75)	175.5 (29)	165.5 (31.25)	179.5 (38)	173.5 (25)	180 (31.5)
Min-Y with closed eyes (mm)	246 (25)	241 (20.5)	239 (8.5)	244.5 (28.75)	246 (14.75)	243.5 (29)
Max-X with closed eyes (mm)	179.5 (25.5)	185 (32.25)	174 (29.25)	186.5 (39.5)	182.5 (21.25)	188.5 (31.75)
Max-Y with closed eyes (mm)	249 (24.25)	246 (21.25)	243.5 (8.75)	247.5 (33.25)	251 (16.75)	248.5 (29)
Distance covered with closed eyes (mm)	61.5 (89.5)	67.50 (64.75)	65.50 (35.75)	70.0 (30.25)	74.50 (44.25)	62.50 (40.5)
Area with closed eyes (mm^2^)	4.0 (4.0)	3.50 (8.00)	3.0 (5.00)	4.5 (3.25)	7.0 (7.0)	3.0 (5.25)

T0: outcome measures at baseline; T1: outcome measures at post-treatment assessment; T2: outcome measures at follow-up assessment.

**Table 3:** Results of repeated measures analysis.

Variables	Intragroup Effect		Time × Group Interaction		
	F	ES	F	ES	Power (1–β)
Dorsiflexion under load	43.73 **	0.46	4.90 *	0.08	0.90
Dorsal flexion in unloading	17.12 **	0.25	3.06	0.05	0.70
Plantar flexion in unloading	18.46 **	0.27	3.78 *	0.07	0.85
Internal malleolus pressure pain threshold	20.62 **	0.29	2.75	0.05	0.70
External malleolus pressure pain threshold	10.41 **	0.17	2.11	0.04	0.59
Min-X with open eyes	1.38	0.05	0.56	0.02	0.32
Min-Y with open eyes	3.87 *	0.13	2.69	0.10	0.95
Max-X with open eyes	2.01	0.07	0.79	0.03	0.46
Max-Y with open eyes	3.80 *	0.13	3.44	0.12	0.98
Distance covered with open eyes	2.12	0.08	0.88	0.03	0.46
Area with open eyes	1.52	0.06	0.71	0.02	0.32
Min-X with closed eyes	1.08	0.04	0.15	0.01	0.17
Min-Y with closed eyes	0.73	0.03	0.81	0.03	0.46
Max-X with closed eyes	2.38	0.09	2.78	0.10	0.95
Max-Y with closed eyes	0.02	0.01	1.91	0.07	0.85
Distance covered with closed eyes	2.75	0.10	0.15	0.01	0.17
Area with closed eyes	0.65	0.03	4.72 *	0.16	0.99

F: Fisher-Snedecor statistic; ES: effect size (η^2^_p_: partial square eta); Power (1–β): Post hoc power calculated with effect size (f). * Significant difference between improvements of the study groups (*p* < 0.05). ** Significant difference between improvements of the study groups (*p* < 0.01).

**Table 4:** Pairwise comparison analysis.

Variables	T1-T0	T2-T1	T2-T0
Dorsiflexion under load	1.69 [1.10; 2.28] (*p* = 0.00)	−0.01 [−0.26; 0.24] (*p* = 1.00)	1.68 [1.06; 2.31] (*p* = 0.00)
Dorsal flexion in unloading	1.76 [0.74; 2.78] (*p* = 0.00)	0.32 [−0.47; 1.12] (*p* = 0.95)	2.08 [1.06; 3.11] (*p* = 0.00)
Plantar flexion in unloading	2.78 [1.03; 4.52] (*p* = 0.00)	−0.89 [−1.79; 0.003] (*p* = 0.05)	2.78 [1.03; 4.52] (*p* = 0.001)
Internal malleolus pressure pain threshold	22.26 [12.87; 31.66] (*p* = 0.00)	−4.21 [−15.14; 6.72] (*p* = 1.00)	18.05 [11.57; 24.53] (*p* = 0.00)
External malleolus pressure pain threshold	13.76 [4.83; 22.69] (*p* = 0.001)	−0.38 [−10.17; 9.41] (*p* = 1.00)	13.38 [6.92; 19.84] (*p* = 0.00)
Min-X with open eyes	−1.36 [−9.86; 7.12] (*p* = 1.00)	−3.26 [−9.88; 3.35] (*p* = 0.64)	−4.63 [−11.46; 2.19] (*p* = 0.28)
Min-Y with open eyes	−7.20 [−13.69; −0.70] (*p* = 0.02)	6.61 [−2.17; 15.41] (*p* = 0.19)	−0.58 [−7.25; 6.09] (*p* = 1.00)
Max-X with open eyes	−2.17 [−12.09; 7.74] (*p* = 1.00)	−4.29 [−11.56; 2.96] (*p* = 0.42)	−6.47 [−14.49; 1.54] (*p* = 0.14)
Max-Y with open eyes	−7.63 [−13.55; −1.71] (*p* = 0.01)	6.03 [−3.11; 15.17] (*p* = 0.30)	−1.59 [−8.71; 5.52] (*p* = 1.00)
Distance covered with open eyes	−2.48 [−14.27; 9.31] (*p* = 1.00)	−8.72 [−21.42; 3.98] (*p* = 0.27)	−11.20 [−29.79; 7.39] (*p* = 0.40)
Area with open eyes	−0.86 [−5.79; 4.07] (*p* = 1.00)	−2.31 [−6.34; 1.72] (*p* = 0.46)	−3.17 [−8.59; 2.24] (*p* = 0.43)
Min-X with closed eyes	4.51 [−8.57; 17.61] (*p* = 1.00)	−6.06 [−18.02; 5.89] (*p* = 0.61)	−1.54 [−8.75; 5.65] (*p* = 1.00)
Min-Y with closed eyes	−1.01 [−12.57; 10.54] (*p* = 1.00)	−8.42 [−32.42; 15.57] (*p* = 1.00)	−9.43 [−36.77; 17.9] (*p* = 1.00)
Max-X with closed eyes	2.00 [−3.77; 7.77] (*p* = 1.00)	−4.98 [−10.58; 0.61] (*p* = 0.09)	−2.98 [−9.34; 3.37] (*p* = 0.71)
Max-Y with closed eyes	0.06 [−12.01; 12.14] (*p* = 1.00)	−0.76 [−8.67; 7.13] (*p* = 1.00)	−0.70 [−11.57; 10.17] (*p* = 1.00)
Distance covered with closed eyes	7.66 [−10.67; 25.99] (*p* = 0.87)	−17.16 [−38.61; 4.28] (*p* = 0.15)	−9.50 [−25.97; 6.97] (*p* = 0.45)
Area with closed eyes	−0.76 [−3.13; 1.60] (*p* = 1.00)	−0.36 [−2.80; 2.06] (*p* = 1.00)	−1.13 [−4.12; 1.84] (*p* = 1.00)

T1-T0: outcome measures for post-treatment to baseline assessments; T2-T1: outcome measures for follow-up to post-treatment assessments; T2-T0: outcome measures for follow-up to baseline assessments.

**Table 5:** Inter-rater reliability coefficients, SEM values, and MDC_95_ estimates for each measured variable.

Measured Variable	ICC	SEM	MDC_95_	n (%)	
				CG	EG
Dorsiflexion under load	0.71	0.22	0.61	5/14 (35.71)	10/12 (83.33)
Dorsal flexion in unloading	0.87	1.07	2.97	4/14 (28.57)	6/12 (50)
Plantar flexion in unloading	0.98	1.65	4.56	4/14 (28.57)	5/12 (41.67)
External malleolus pressure pain threshold	0.94	2.97	8.23	4/14 (28.57)	4/12 (33.33)
Internal malleolus pressure pain threshold	0.64	10.36	28.7	7/14 (50)	6/12 (50)
Area with open eyes	0.56	0.02	0.07	4/14 (28.57)	4/12 (33.33)
Area with closed eyes	0.77	0.04	0.12	4/14 (28.57)	2/12 (16.67)

ICC: Intraclass Correlation Coefficient; SEM: Standard Error of Measurement; MDC_95_: Minimum Detectable Change at 95% confidence; n (%): Patients with Clinically Relevant Improvement (percentage); CG: control group; EG: experimental group.

## Data Availability

The data that support the findings of this study are available on request from the corresponding author.
